# Supplementary material for: Mutations in PCYT2 disrupt etherlipid biosynthesis and cause a complex hereditary spastic paraplegia
Source: Brain. 2019 Oct 22;142(11):3382–97. doi: 10.1093/brain/awz291 (PMC6821184; doi:10.1093/brain/awz291)
Supplement: awz291_Supplementary_Data [file awz291_supplementary_data.zip › awz291-Suppl_data/Supplementary_Data4.zip!/Supplementary information 4 - plasma lipidomics.zip!/Plasma lipidomics/Control vs Patient2/Statistical plots and data/SPH species/Boxplots.pdf]

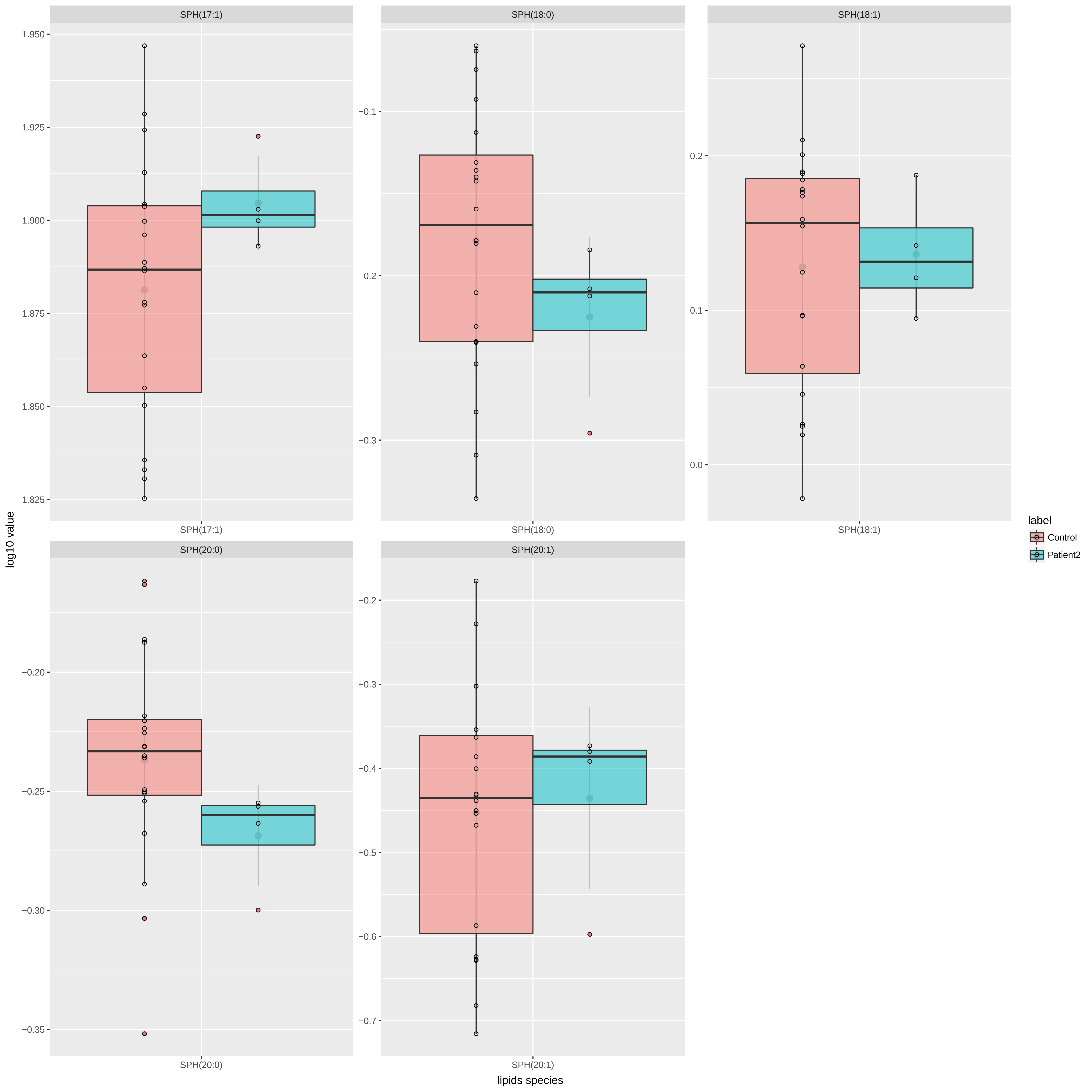
SPH(17:1)
SPH(18:0)
SPH(18:1)
SPH(20:0)
SPH(20:1)
1.950
1.925
1.900
1.875
1.850
1.825
−0.1
−0.2
−0.3
0.2
0.1
0.0
−0.20
−0.25
−0.30
−0.35
−0.2
−0.3
−0.4
−0.5
−0.6
−0.7
log10 value
lipids species
label
Control
Patient2
